# SoSyM reflections: the 2021 "state of the journal" report

**DOI:** 10.1007/s10270-022-00979-1

**Published:** 2022-02-07

**Authors:** Huseyin Ergin, Jeff Gray, Bernhard Rumpe, Martin Schindler

**Affiliations:** 1 Ball State University, Muncie, USA; 2 University of Alabama, Tuscaloosa, AL USA; 3 RWTH Aachen University, Aachen, Germany

The inaugural issue of each SoSyM volume-year is usually written during a time of personal and professional reflection. Over the past 12 months, researchers from all disciplines continued to experience the need for flexibility as the COVID-19 pandemic extended its impact across the globe through many variants. This has forced us all to adapt in many ways such that the idea of “virtual everything” permeates our daily discussions. The software and systems modeling community continued to thrive and exhibited a great degree of flexibility and increased production in the overall research output, despite the limitations for in-person collaborations. In fact, for many years, the first issue of each SoSyM volume-year has reported growth in submissions and other important metrics. The same is true for the 2021 publication year of SoSyM. The journal continues a positive and healthy trajectory in the increased number of submissions and an impact factor that ranks SoSyM among the top publication venues for software engineering.

The following sections report on various statistics and general updates about SoSyM that occurred over 2021. We invite all authors to continue submitting their contributions to SoSyM, and we are always available to correspond regarding questions about the suitability of an idea or potential submission. We wish you all of the best for a productive and satisfying year of research and personal growth in 2022!

## 2021 Summary statistics

The six SoSyM issues published in 2021 contained 40 Regular papers, 32 Special Section papers, 5 Theme Section papers, 4 Expert Voices, 6 Guest Editorials, and 1 Erratum. In total, 2197 pages were published in volume 20. This is a 38% increase compared to the previous year and continues to represent the commitment by our publisher (Springer) in reducing the time to publication by processing papers expeditiously after acceptance. We are grateful to Elizabeth Dziubela, our Springer liaison, for her helpful efforts in assisting us with the expansion.

We are excited to report that the 2-year impact factor (IF) for SoSyM remains very respectable at 1.910 (previously at 1.876 in 2020 and 2.66 in the record year of 2019). The 5-year IF increased to 2.074 (from 1.915 last year). Furthermore, the h-5 Google Scholar ranking places SoSyM at #14 among all conferences and journals related to software engineering and programming languages. Further rankings can be found at https://www.sosym.org/.

We have observed that modeling continues to develop in overall maturity across the core domains of Software Engineering and Information Systems. Additionally, there is a growing interest in SoSyM among researchers who focus on model-based/model-driven activities in a wider area of software and systems engineering. This includes man-made systems, such as cars, airplanes, cell phones, and health care devices, but also existing systems from nature, which include biological systems, chemical structures and interactions, and of course complex physical or medical activities in various areas. Thus, it was natural that the number of submissions increased in 2021 over previous years. Over the past year, SoSyM received 420 submissions—more than 50 additional submissions compared to 2020 and the largest number for any year in our history. Also, the number of downloads increased again. At the end of 2020, there were 179,555 downloaded SoSyM articles during the calendar year. Comparatively, the final 2021 yearly download total was 197,730.

The acceptance rate increased in 2021 to 34.9% which seems to be coming from the fact that SoSyM has a strong reputation and the number of low-quality papers submitted has considerably decreased. Unfortunately, the average time from submission to the final decision (accept or reject) has also increased to 170 days (146 days in 2020). It remains a challenge for our Editors to identify reviewers amid a community that has the specific expertise in areas covered by SoSyM and the increased submissions are starting to exhibit new challenges in the decision time.

## SoSyM's 10-year most influential paper awards

When a research community matures, it is often interesting to take a look back through history to observe what contributions had the most impact and what topics emerged as most prominent over specific periods of time. Our collaboration with the MODELS conference has provided an opportunity for us to honor the authors of the most influential papers in our community. Each year, SoSyM identifies the two papers (from the Regular and Theme Section areas) that had the most impact over the past decade since their publication. The selection is based on the ISI citation index among papers published in SoSyM since 2010. The following two papers were presented virtually at MODELS 2021, and each author received an award certificate. We congratulate the authors for these “Most Influential” papers of SoSyM over the past decade.

The *SoSyM 2021 "10-year most influential Regular paper award"* was given to:

Zoltán Micskei and Hélène Waeselynck, "The many meanings of UML 2 Sequence Diagrams: A survey", In: *Journal on Software and* Systems *Modeling (SoSyM)*, Volume 10, Issue 4, pp. 489–514, Springer, October 2011.

https://doi.org/10.1007/s10270-010-0157-9

The *SoSyM 2021 "10-year most influential Theme Section paper award"* was given to:

Simona Bernardi, José Merseguer, and Dorina C. Petriu, "A dependability profile within MARTE", In: *Journal on Software and Systems Modeling (SoSyM)*, Volume 10, Issue 3, pp. 313–336, Springer, July 2011.

https://doi.org/10.1007/s10270-009-0128-1

More information about the awards can be found at: http://www.sosym.org/awards/.

## SoSyM's journal-first papers at MODELS 2021

The collaboration between SoSyM and the MODELS conference continued with the organization of the SoSyM “Journal-First” option. This collaboration enables authors of recent SoSyM papers to present their work across the core conference sessions at MODELS. Through this collaboration, SoSyM authors have the opportunity to reach a broader audience to present their work. This includes research talks that explore more depth through analytical and empirical evidence than found in a typical MODELS conference paper. At MODELS 2021, a record number of “SoSyM First” papers were presented (30 papers). We are very thankful to the MODELS 2021 PC Chairs, Shiva Nejati and Daniel Varro, for their help in the integration of the SoSyM First papers into the general MODELS 2021 schedule. The SoSyM First papers presented at MODELS 2021 were the following (note: Some of the papers are available online but have not yet received an assignment to an issue; some of the papers listed below also appear in this same issue):

- Moussa Amrani, Dominique Blouin, Robert Heinrich, Arend Rensink, Hans Vangheluwe, and Andreas Wortmann, "Multi-paradigm modelling for cyber–physical systems: A descriptive framework", In: *Journal on Software and Systems Modeling (SoSyM)*, Volume 20, Issue 3, pp. 611–639, Springer, June 2021. https://doi.org/10.1007/s10270-021-00876-z
- Sebastian Pilarski, Martin Staniszewski, Matthew Bryan, Frederic Villeneuve, and Dániel Varró, "Predictions-on-chip: model-based training and automated deployment of machine learning models at runtime", In: *Journal on Software and Systems Modeling (SoSyM)*, Volume 20, Issue 3, pp. 685–709, Springer, June 2021. https://doi.org/10.1007/s10270-020-00856-9
- Ferenc A. Somogyi, Gergely Mezei, Zoltán Theisz, Sándor Bácsi, and Dániel Palatinszky, "Playground for multi-level modeling constructs", In: *Journal on Software and Systems Modeling (SoSyM)*, Springer, in press, 2021. https://doi.org/10.1007/s10270-021-00900-2
- Adrien Le Coënt, Julien Alexandre dit Sandretto, and Alexandre Chapoutot, "Guaranteed master for interval-based cosimulation", In: *Journal on Software and Systems Modeling (SoSyM)*, Volume 20, Issue 3, pp. 711–724, Springer, June 2021. https://doi.org/10.1007/s10270-020-00858-7
- Léa Brunschwig, Esther Guerra, and Juan de Lara, "Modelling on mobile devices—A systematic mapping study", In: *Journal on Software and Systems Modeling (SoSyM)*, Springer, in this issue, 2021. https://doi.org/10.1007/s10270-021-00897-8
- Saša Kuhar and Gregor Polancic, "Conceptualization, measurement, and application of semantic transparency in visual notations—A systematic literature review", In: *Journal on Software and Systems Modeling (SoSyM)*, Volume 20, Issue 6, pp. 2155–2197, Springer, December 2021. https://doi.org/10.1007/s10270-021-00888-9
- Walter Cazzola, Sudipto Ghosh, Mohammed Al-Refai, and Gabriele Maurina, "Bridging the model-to-code abstraction gap with fuzzy logic in model-based regression test selection", In: *Journal on Software and Systems Modeling (SoSyM)*, Springer, in this issue, 2021. https://doi.org/10.1007/s10270-021-00899-6
- Gábor Bergmann, "Controllable and decomposable multidirectional synchronizations", In: *Journal on Software and Systems Modeling (SoSyM)*, Volume 20, Issue 5, pp. 1735–1774, Springer, October 2021. https://doi.org/10.1007/s10270-021-00879-w
- Javier Troya, Nathalie Moreno, Manuel F. Bertoa, and Antonio Vallecillo, "Uncertainty representation in software models: A survey", In: *Journal on Software and Systems Modeling (SoSyM)*, Volume 20, Issue 4, pp. 1183–1213, Springer, August 2021. https://doi.org/10.1007/s10270-020-00842-1
- Lissette Almonte, Esther Guerra, Iván Cantador, and Juan de Lara, "Recommender systems in model-driven engineering—A systematic mapping review", In: *Journal on Software and Systems Modeling (SoSyM)*, Springer, in this issue, 2021. https://doi.org/10.1007/s10270-021-00905-x
- Martina De Sanctis, Ludovico Iovino, Maria Teresa Rossi, and Manuel Wimmer, "MIKADO: A smart city KPIs assessment modeling framework", In: *Journal on Software and Systems Modeling (SoSyM)*, Springer, in this issue, 2021. https://doi.org/10.1007/s10270-021-00907-9
- Wenjun Xiong, Emeline Legrand, Oscar Åberg, and Robert Lagerström, "Cyber security threat modeling based on the MITRE Enterprise ATT&CK Matrix", In: *Journal on Software and Systems Modeling (SoSyM)*, Springer, in this issue, 2021. https://doi.org/10.1007/s10270-021-00898-7
- Alexander Boll, Florian Brokhausen, Tiago Amorim, Timo Kehrer, and Andreas Vogelsang, "Characteristics, potentials, and limitations of open-source Simulink projects for empirical research", In: *Journal on Software and Systems Modeling (SoSyM)*, Volume 20, Issue 6, pp. 2111–2130, Springer, December 2021. https://doi.org/10.1007/s10270-021-00883-0
- Oszkár Semeráth, Aren A. Babikian, Boqi Chen, Chuning Li, Kristóf Marussy, Gábor Szárnyas, and Dániel Varró, "Automated generation of consistent, diverse and structurally realistic graph models", In: *Journal on Software and Systems Modeling (SoSyM)*, Volume 20, Issue 5, pp. 1713–1734, Springer, October 2021. https://doi.org/10.1007/s10270-021-00884-z
- Stéphanie Challita, Fabian Korte, Johannes Erbel, Faiez Zalila, Jens Grabowski, and Philippe Merle, "Model-based cloud resource management with TOSCA and OCCI", In: *Journal on Software and Systems Modeling (SoSyM)*, Volume 20, Issue 5, pp. 1609–1631, Springer, October 2021. https://doi.org/10.1007/s10270-021-00869-y
- Shahar Maoz and Jan Oliver Ringert, "Spectra: A specification language for reactive systems", In: *Journal on Software and Systems Modeling (SoSyM)*, Volume 20, Issue 5, pp. 1553–1586, Springer, October 2021. https://doi.org/10.1007/s10270-021-00868-z
- Swaib Dragule, Thorsten Berger, Claudio Menghi, and Patrizio Pelliccione, "A survey on the design space of end-user-oriented languages for specifying robotic missions", In: *Journal on Software and Systems Modeling (SoSyM)*, Volume 20, Issue 4, pp. 1123–1158, Springer, August 2021. https://doi.org/10.1007/s10270-020-00854-x
- Weslley Torres, Mark G. J. van den Brand, and Alexander Serebrenik, "A systematic literature review of cross-domain model consistency checking by model management tools", In: *Journal on Software and Systems Modeling (SoSyM)*, Volume 20, Issue 3, pp. 897–916, Springer, June 2021. https://doi.org/10.1007/s10270-020-00834-1
- Pablo Gómez-Abajo, Esther Guerra, Juan de Lara, and Mercedes G. Merayo, "Wodel-Test: A model-based framework for language-independent mutation testing", In: *Journal on Software and Systems Modeling (SoSyM)*, Volume 20, Issue 3, pp. 767–793, Springer, June 2021. https://doi.org/10.1007/s10270-020-00827-0
- Jaime Font, Lorena Arcega, Øystein Haugen, and Carlos Cetina, "Handling nonconforming individuals in search-based model-driven engineering: nine generic strategies for feature location in the modeling space of the meta-object facility", In: *Journal on Software and Systems Modeling (SoSyM)*, Volume 20, Issue 5, pp. 1653–1688, Springer, October 2021. https://doi.org/10.1007/s10270-021-00870-5
- Sina Madani, Dimitris Kolovos, and Richard F. Paige, "Distributed model validation with Epsilon", In: *Journal on Software and Systems Modeling (SoSyM)*, Volume 20, Issue 5, pp. 1689–1712, Springer, October 2021. https://doi.org/10.1007/s10270-021-00878-x
- Maher Fakih, Oliver Klemp, Stefan Puch, and Kim Grüttner, "A modeling methodology for collaborative evaluation of future automotive innovations", In: *Journal on Software and Systems Modeling (SoSyM)*, Volume 20, Issue 5, pp. 1587–1608, Springer, October 2021. https://doi.org/10.1007/s10270-021-00864-3
- Mahsa Panahandeh, Mohammad Hamdaqa, Bahman Zamani, and Abdelwahab Hamou-Lhadj, "MUPPIT: A method for using proper patterns in model transformations", In: *Journal on Software and Systems Modeling (SoSyM)*, Volume 20, Issue 5, pp. 1491–1523, Springer, October 2021. https://doi.org/10.1007/s10270-020-00853-y
- Siamak Farshidi, Slinger Jansen, and Sven Fortuin, "Model-driven development platform selection: Four industry case studies", In: *Journal on Software and Systems Modeling (SoSyM)*, Volume 20, Issue 5, pp. 1525–1551, Springer, October 2021. https://doi.org/10.1007/s10270-020-00855-w
- Arvind Nair, Xia Ning, and James H. Hill, "Using recommender systems to improve proactive modeling", In: *Journal on Software and Systems Modeling (SoSyM)*, Volume 20, Issue 4, pp. 1159–1181, Springer, August 2021. https://doi.org/10.1007/s10270-020-00841-2
- David Granada, Juan M. Vara, Mercedes Merayo, and Esperanza Marcos, "CEViNEdit: Improving the process of creating cognitively effective graphical editors with GMF", In: *Journal on Software and Systems Modeling (SoSyM)*, Volume 20, Issue 3, pp. 867–895, Springer, June 2021. https://doi.org/10.1007/s10270-020-00833-2
- Matias Pol'la, Agustina Buccella, and Alejandra Cechich, "Analysis of variability models: A systematic literature review", In: *Journal on Software and Systems Modeling (SoSyM)*, Volume 20, Issue 4, pp. 1043–1077, Springer, August 2021. https://doi.org/10.1007/s10270-020-00839-w
- Feng Zhu and Jun Tang, "Graphical composite modeling and simulation for multi-aircraft collision avoidance", In: *Journal on Software and Systems Modeling (SoSyM)*, Volume 20, Issue 3, pp. 821–835, Springer, June 2021. https://doi.org/10.1007/s10270-020-00830-5
- Stefan Klikovits and Didier Buchs, "Pragmatic reuse for DSML development", In: *Journal on Software and Systems Modeling (SoSyM)*, Volume 20, Issue 3, pp. 837–866, Springer, June 2021. https://doi.org/10.1007/s10270-020-00831-4
- Mojtaba Bagherzadeh, Karim Jahed, Benoit Combemale, and Juergen Dingel, "Live modeling in the context of state machine models and code generation", In: *Journal on Software and Systems Modeling (SoSyM)*, Volume 20, Issue 3, pp. 795–819, Springer, June 2021. https://doi.org/10.1007/s10270-020-00829-y

## Changes to the editorial board

Jon Whittle is pursuing interesting new opportunities in his position as director of Australia’s national science agency, CSIRO Data61. His new focus spans many different disciplines of scientific inquiry. Jon stepped down from the SoSyM Editorial Board this year after nearly 20 years of service to the software and systems modeling community. We are grateful for his assistance and wish him all of the best in his new professional endeavors.
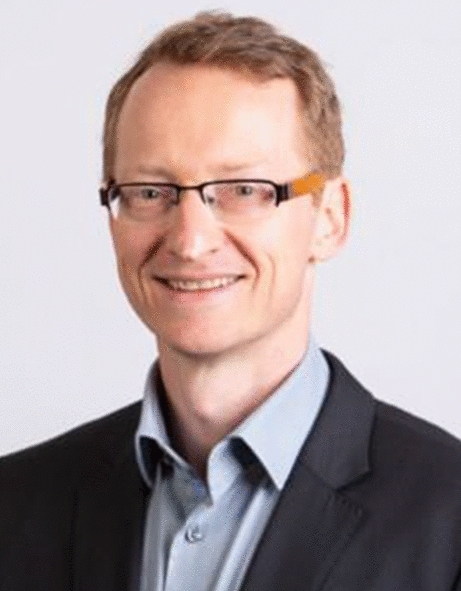


### Reviewers in 2021

A strong research community depends on the efforts of volunteers who help serve as reviewers. The software and systems modeling community has always risen to the request for help from SoSyM. We appreciate all of the help that the reviewers provided in service to the modeling community! We would also like to offer special recognition to the following reviewers, who were recommended as the SoSyM Best Reviewers of 2021, based on the technical depth and feedback provided to authors over the past year—congratulations! Each of the following reviewers received a certificate of recognition:

Colin Atkinson, Dominik Bork, Erwan Bousse, Travis Breaux, Carlos Cuesta, Martina De Sanctis, Juergen Dingel, Dirk Fahland, Loic Helouet, Nicolas Hili, Jörg Holtmann, Jennifer Horkoff, Thomas Kühne, Akhil Kumar, Xavier Le Pallec, Assaf Marron, Bentley Oakes, Anthony Simons, Thomas Vogel, and Marco Wehrmeister.

Below is a list of those who reviewed one or more papers for the journal in the last year. The complete list of reviewers can also be found on our website http://www.sosym.org/people/.

Mohamed Abdelrazek, Suraj Ajit, Omar Alam, Ian Alexander, Hessa Alfraihi, Shaukat Ali, Joao Paulo Almeida, Ahmad Salim Al-Sibahi, Sanaa Alwidian, Vasco Amaral, Daniel Amyot, Paolo Arcaini, Wesley K. G. Assuncao, Colin Atkinson, Joanne Atlee, Claudia Ayala, Onder Babur, Omar Badreddin, Mira Balaban, Arosha Bandara, Torsten Bandyszak, Olivier Barais, Mikhail Barash, Luciano Baresi, Ankica Barisic, Konstantinos Barmpis, Jorge Barreiros, Angela Barriga, Thais Batista, Edouard Batot, Dinesh Batra, Steffen Becker, Nelly Bencomo, Luca Berardinelli, Robin Bergenthum, Thorsten Berger, Gabor Bergmann, Ilia Bider, Gordon Blair, Dominique Blouin, Alvine Boaye-Belle, Francis Bordeleau, Dominik Bork, Artur Boronat, Paolo Bottoni, Juan Boubeta-Puig, Johann Bourcier, Erwan Bousse, Jonathan Bowen, Drazen Brdjanin, Travis Breaux, Isabel Brito, Jan Broenink, Jean-Michel Bruel, Davide Brugali, Hugo Bruneliere, Alessio Bucaioni, Antonio Bucchiarone, Robert Buchmann, Thomas Buchmann, Andrea Burattin, Erik Burger, Loli Burgueño, Rimantas Butleris, Cristina Cabanillas, Jordi Cabot, Javier Luis Canovas Izquierdo, Rafael Capilla, Emerson Carneiro de Andrade, Victorio Carvalho, Kārlis Čerāns, Carlos Cetina, Moharram Challenger, Michel Chaudron, Xin Chen, Antonio Cicchetti, Federico Ciccozzi, Robert Clarisó, Tony Clark, Manuel Clavel, Jane Cleland-Huang, Loek Cleophas, Benoit Combemale, Carlo Combi, José Conejero, Carl Corea, Dolors Costal, Jesús Sánchez Cuadrado, Carlos Cuesta, Alcino Cunha, Alberto da Silva, Fabiano Dalpiaz, Andrea D'Ambrogio, Nancy Day, Jose Luis de la Vara, Juan de Lara, Martina De Sanctis, Pierre De Saqui-Sannes, Marne de Vries, Julien DeAntoni, Renzo Degiovanni, Thomas Degueule, Joerg Desel, Byron DeVries, Xavier Devroey, Claudio Di Ciccio, Juri Di Rocco, Davide Di Ruscio, Vasiliki Diamantopoulou, Marcos Didonet Del Fabro, Juergen Dingel, Julio do Prado Leite, Marlon Dumas, Francisco Duran, Johann Eder, Alexander Egyed, Neil Ernst, Bedilia Estrada, Dirk Fahland, Kleinner Farias, Michael Fellmann, João Miguel Fernandes, Eduardo Fernandez, Nicolas Ferry, Robson Fidalgo, Hans-Georg Fill, Xavier Franch, Ulrich Frank, Marc Frappier, Piero Fraternali, Walid Gaaloul, Kelly Garces, Felix Garcia, Luciano Garcia Banuelos, Antonio García-Domínguez, Ning Ge, Sebastien Gerard, Sepideh Ghanavati, Mohamad Gharib, Aditya Ghose, Holger Giese, Christophe Gnaho, Sebastian Goetz, Arda Goknil, Thomas Goldschmidt, Cláudio Gomes, Abel Gómez, Elena Gómez-Martínez, Miguel Goulão, Catarina Gralha, David Granada, Joel Greenyer, Paul Grefen, Georg Grossmann, Eoin Grua, John Grundy, Giancarlo Guizzardi, Renata Guizzardi, Sarra Habchi, Simon Hacks, Irit Hadar, Nabil Hameurlain, Regina Hebig, Monika Heiner, Robert Heinrich, Maritta Heisel, Nicolas Hili, Georg Hinkel, Knut Hinkelmann, Frank Hogrebe, Jörg Holtmann, Stijn Hoppenbrouwers, Jennifer Horkoff, Marianne Huchard, John Hutchinson, Emilio Insfran, Ludovico Iovino, Muhammad Zohaib Iqbal, Fuyuki Ishikawa, Ana Ivanchikj Moroni, Amin Jalali, Dietmar Jannach, Axel Jantsch, Steve Jenkins, Manfred A. Jeusfeld, Mohamed Jmaiel, Paul Johannesson, Reyes Juarez-Ramirez, Nafiseh Kahani, Jānis Kampars, Geylani Kardas, Gabor Karsai, Timo Kehrer, Steven Kelly, Wael Kessentini, Djamel Eddine Khelladi, Marite Kirikova, Alexander Knapp, Shekoufeh Kolahdouz-Rahimi, Dimitrios Kolovos, Dimitris Kolovos, Anil Koyuncu, Lars Michael Kristensen, Thomas Kuehn, Thomas Kühne, Géza Kulcsár, Vinay Kulkarni, Akhil Kumar, Yvan Labiche, Katsiaryna Labunets, Robert Lagerström, Leen Lambers, Elyes Lamine, Yngve Lamo, Kevin Lano, Xavier Le Pallec, Edward Lee, Martti Lehto, Henrik Leopold, Timothy Lethbridge, Nianyu Li, Shuai Li, Tong Li, Grischa Liebel, Crescencio Lima, Igor Linkov, Patricia Lopez, Víctor López-Jaquero, Oscar Luis, Roman Lukyanenko, Mass Soldal Lund, Xiaoxing Ma, Nuno Macedo, Fernando Macías, Paulo Maia, Mahdi Manesh, Beatriz Marín, Assaf Marron, Salvador Martinez, Nicholas Matragkas, Raimundas Matulevicius, Davide Andrea Mauro, Julio Medina, Anna Medve, Claudio Menghi, Giovanni Meroni, Jose Merseguer, Judith Michael, Fredrik Milani, Mark Minas, Miguel Mira da Silva, Pieter Mosterman, Hassan Mountassir, Haralambos Mouratidis, Saad Mubeen, Paula Muñoz, John Mylopoulos, Elisa Yumi Nakagawa, Elena Navarro, Lukas Netz, Bernd Neumayr, Phu Nguyen, Phuong Nguyen, Nan Niu, Arne Nordmann, Bentley Oakes, Ileana Ober, Johnny Öberg, Edson OliveiraJr, Alessandro Oltramari, Xavier Oriol, Michiel Overeem, Richard Freeman Paige, Elda Paja, Liliana Pasquale, Oscar Pastor, Cecile Péraire, Francisca Pérez, Robert Pettit, Alfonso Pierantonio, Joao Pimentel, Monica Pinto, Stephan Poelmans, Geert Poels, Pascal Poizat, Andrea Polini, Saheed Popoola, Pasqualina Potena, Henderik Proper, Truong Ho Quang, Elisa Quintarelli, Ansgar Radermacher, Akshay Rajhans, Jolyta Ralyté, Qusai Ramadan, Nacim Ramdani, Aurora Ramírez, Eric J. Rapos, Alexander Raschke, Gil Regev, Manfred Reichert, Hajo Reijers, Elvinia Riccobene, Jan Oliver Ringert, Erkuden Rios Velasco, Roberto Rodríguez-Echeverría, Michael Rosemann, Pedro Rossel, Ivan Ruchkin, Marcela Ruiz, Adrian Rutle, Mehrdad Saadatmand, Mahsa Sadi, Andrey Sadovykh, Neda Saeedloei, Houari Sahraoui, Rijul Saini, Mattia Salnitri, Leila Samimi, Jesús Sánchez Cuadrado, Kurt Sandkuhl, Stefan Sauer, Clemens Sauerwein, Christian Schilling, David Schmalzing, David Schmelter, Rainer Schmidt, Stefan Schönig, Stefan Schulte, Ulrik Schultz, Christoph Schütz, Ed Seidewitz, Ronny Seiger, Bran Selic, Laura Semini, Sagar Sen, Arik Senderovich, Estefanía Serral Asensio, Mojtaba Shahin, Ramy Shahin, Mohammadreza Sharbaf, Carla Silva, Anthony Simons, Monique Snoeck, Pnina Soffer, Oleg Sokolsky, Hui Song, Wei Song, Jean-Sebastien Sottet, Thomas Springer, Emmanouela Stachtiari, Matthew Stephan, Perdita Stevens, Janis Stirna, Ketil Stølen, Volker Stolz, Daniel Strüber, Arnon Sturm, Allison Sullivan, Yu Sun, Daniel Sundmark, Gerson Sunye, Angelo Susi, Andreas Symeonidis, Eugene Syriani, Gabriele Taentzer, Jérémie Tatibouet, Paul Temple, Ernest Teniente, Thomas Thüm, Matthias Tichy, Ulyana Tikhonova, Massimo Tisi, Juha-Pekka Tolvanen, Victoria Torres, Konstantinos Traganos, Hanh Nhi Tran, Marina Tropmann-Frick, Javier Troya, Christos Tsigkanos, Katja Tuma, Samuel Tweneboah-Koduah, Mark Utting, Antonio Vallecillo, Nick R. T. P. van Beest, Mark van den Brand, Han van der Aa, Wil M.P. van der Aalst, Tijs van der Storm, Jean Vanderdonckt, Irene Vanderfeesten, Hans Vangheluwe, Juan Manuel Vara, Daniel Varro, Eric Verbeek, Eugenio Villar, Thomas Vogel, Johannes von Oswald, Yves Wautelet, Barbara Weber, Thomas Weber, Marco Wehrmeister, Ran Wei, Nils Weidmann, Hans Weigand, Mathias Weske, Bernhard Westfechtel, Martin Weyssow, Manuel Wimmer, Genta Indra Winata, Carson Woo, Andreas Wortmann, Franz Wotawa, Sebastian Wrede, Jianqing Wu, Wenhua Yang, Bahman Zamani, Anna Zamansky, Jelena Zdravkovic, Philipp Zech, Bernard P. Zeigler, Lingfang Zeng, Peng Zeng, Li Zhang, Man Zhang, Nan Zhang, Haiyan Zhao, Athanasios Zolotas, Steffen Zschaler, and Albert Zuendorf.

## Contents of this issue

This issue also includes a special Expert Voice article, with the thought provoking title, *“Models: The fourth dimension of computer science—Towards studies of models and modelling,”* contributed by Bernhard Thalheim. Bernhard is a long-time member of our community, and his paper contains many insights and action points for your consideration. We strongly recommend his paper for your consideration and reflection on the role of modeling in computer science.

The contents of this issue are as follows:

1. **Expert voice**
2. “Models: The fourth dimension of computer science—Towards studies of models and modelling” by Bernhard Thalheim
3. **Overview paper**
4. "Model-driven engineering for mobile robotic systems: A systematic mapping study" by Giuseppina Casalaro, Giulio Cattivera, Federico Ciccozzi, Ivano Malavolta, Andreas Wortmann, and Patrizio Pelliccione
5. **Regular papers**
6. "On the automation-supported derivation of domain-specific UML profiles considering static semantics" by Alexander Kraas
7. "Suggesting model transformation repairs for rule-based languages using a contract-based testing approach" by Roberto Rodríguez-Echeverría, Fernando Macías, Adrian Rutle, and José Conejero
8. "An ontological metamodel for cyber-physical system safety, security, and resilience coengineering" by Georgios Bakirtzis, Tim Sherburne, Stephen Adams, Barry Horowitz, Peter Beling, and Cody Fleming
9. "A generic LSTM neural network architecture to infer heterogeneous model transformations" by Loli Burgueño, Jordi Cabot, Shuai Li, and Sebastien Gerard
10. "Cyber security threat modeling based on the MITRE Enterprise ATT&CK Matrix" by Wenjun Xiong, Emeline Legrand, Oscar Åberg, and Robert Lagerström
11. "Modelling on mobile devices—A systematic mapping study" by Léa Brunschwig, Esther Guerra, and Juan de Lara
12. "Bridging the model-to-code abstraction gap with fuzzy logic in model-based regression test selection" by Walter Cazzola, Sudipto Ghosh, Mohammed Al-Refai, and Gabriele Maurina
13. "Guiding the evolution of product-line configurations" by Michael Nieke, Gabriela Sampaio, Thomas Thüm, Christoph Seidl, Leopoldo Teixeira, and Ina Schaefer
14. "Recommender systems in model-driven engineering—A systematic mapping review" by Lissette Almonte, Esther Guerra, Iván Cantador, and Juan de Lara
15. "MIKADO: A smart city KPIs assessment modeling framework" by Martina De Sanctis, Ludovico Iovino, Maria Rossi, and Manuel Wimmer
16. "A method for transforming knowledge discovery metamodel to ArchiMate models" by Ricardo Pérez-Castillo, Andrea Delgado, Francisco Ruiz, Virginia Bacigalupe, and Mario Piattini
17. "Efficient model similarity estimation with robust hashing" by Salvador Martinez, Sebastien Gerard, and Jordi Cabot
18. "Graphic modeling in Distributed Autonomous and Asynchronous Automata (DA3)" by Wiktor Daszczuk
19. "Enhancing software model encoding for feature location approaches based on machine learning techniques" by Ana Cristina Marcén, Francisca Pérez, Oscar Pastor, and Carlos Cetina

We wish you a Happy New Year with the hope that you enjoy reading the papers in this issue!

Huseyin Ergin, Jeff Gray, Bernhard Rumpe, and Martin Schindler.

